# Steroids prevent early recurrence of atrial fibrillation following catheter ablation: a systematic review and meta-analysis

**DOI:** 10.1042/BSR20180462

**Published:** 2018-10-15

**Authors:** Ming Lei, Mengqi Gong, George Bazoukis, Konstantinos P. Letsas, Panagiotis Korantzopoulos, Guangping Li, Gianluigi Bisleri, Benedict Glover, Ka Hou Christien Li, Gary Tse, Adrian Baranchuk, Tong Liu

**Affiliations:** 1 Tianjin Key Laboratory of Ionic-Molecular Function of Cardiovascular Disease, Department of Cardiology, Tianjin Institute of Cardiology, Second Hospital of Tianjin Medical University, Tianjin 300211, P.R. China; 2 Second Department of Cardiology, Laboratory of Cardiac Electrophysiology, Evangelismos General Hospital of Athens, Athens 10676, Greece; 3 First Department of Cardiology, University of Ioannina Medical School, Ioannina, Greece; 4 Division of Cardiac Surgery, Queen’s University, Kingston General Hospital, Kingston, Canada; 5 Division of Cardiology, Kingston General Hospital, Queen’s University, Kingston, Ontario, Canada; 6 Li Ka Shing Institute of Health Sciences, Faculty of Medicine, Chinese University of Hong Kong, Hong Kong, SAR, P.R. China; 7 Department of Medicine and Therapeutics, Faculty of Medicine, Chinese University of Hong Kong, Hong Kong, SAR, P.R. China

**Keywords:** atrial fibrillation, radiofrequency catheter ablation, recurrence, steroids

## Abstract

Previous studies have reported that steroids may reduce the risk of atrial fibrillation (AF) recurrence after catheter ablation, but data regarding this issue have been controversial. Therefore, we conducted a meta-analysis of randomized clinical trials (RCTs) and observational studies to ascertain the association of steroids and AF recurrence after ablation. PubMed, Embase, and Cochrane online databases were searched from inception to December 2017. The primary outcome of the meta-analysis was short-term or long-term AF recurrence following a single ablation procedure with or without the use of steroids. Both fixed- and random-effects models were used to calculate the overall effect estimates. Eight studies (four RCTs and four observational studies), with a total 992 patients, were included in the present study. Our meta-analysis shows that steroid use was associated with reduced AF occurrence at 3 months (odd ratio (OR) = 0.53, 95% confidence interval (CI) = 0.31–0.90, *P*=0.02) and 12–14 months (OR = 0.67, 95% CI = 0.47–0.95, *P*=0.02) after radiofrequency (RF) catheter ablation (RFCA). No clear benefit was observed for AF recurrence at 2–3 days, 1 or 24 months of follow-up. Steroid use was associated with decreased risk of early AF recurrence 3 and 12–14 months after ablation. No clear relationship was observed for 2–3 days, 1 and 24 months of follow-up and further data are needed to clarify these results.

## Introduction

Atrial fibrillation (AF) is the commonest cardiac rhythm disorder observed in clinical practice but it is challenging to cure [1,2]. Radiofrequency (RF) catheter ablation (RFCA) is an effective treatment for drug-refractory symptomatic paroxysmal or persistent AF patients. However, there are still opportunities for recurrence of AF, especially within the first few weeks after ablation [3]. The relationship between inflammation process and the development of AF has been widely studied [4,5].

Recent study has found that the early recurrence of AF after ablation is associated with an inflammatory response to the ablation itself [6]. Halonen et al. [7] showed that hydrocortisone can reduce the incidence of AF after cardiac surgery by preventing inflammatory reactions. Some studies suggested that treatment with steroids after AF ablation is effective for negating AF recurrence by preventing post-ablation inflammatory responses, whereas others have reported no significant benefit. Moreover, the effectiveness of steroids for prevention of AF recurrence may vary at different time points after ablation [8–15]. Given these conflicting findings, we conducted a systematic review and meta-analysis of randomized clinical trials (RCTs) and observational studies to ascertain the impact of steroids on preventing AF recurrence after RFCA.

## Methods

### Search strategy

PubMed, Embase, and Cochrane online databases were searched from their inception to December 2017. The following search string: (hormone or steroid or corticosteroid or glucocorticoid or hydrocortisone or methylprednisolone) and (‘radiofrequency ablation’ or ‘pulmonary vein isolation’ or ‘catheter ablation’ or ‘radiofrequency catheter ablation’) and ‘atrial fibrillation’ were used to identify all the published articles. Searches were not restricted by language. Reference lists of retrieved articles were scanned manually for additional studies.

### Study selection

Inclusion criteria were: (i) the study design was randomized controlled trials (RCTs) or cohort studies, (ii) human subjects, (iii) included the characteristics of study patients, (iv) assessed the effect of steroids on AF recurrence after RFCA. Two reviewers (M.L. and M.G.) independently identified duplicates, and then they conducted the initial screening of all titles or abstracts and evaluated all potentially relevant articles based on full-text reviews. Any disagreements between the two investigators were resolved by discussion with a senior reviewer (T.L.).

### Quality evaluation

In order to limit the heterogeneity secondary to differences amongst study designs, the quality of each study was evaluated to exclude those studies classified as ‘low quality’. This meta-analysis included four RCT studies and four cohort studies. The quality of observational study was assessed using the Newcastle–Ottawa scale (NOS), and NOS ≥ 7 was considered as a high-quality study. We used Cochrane bias risk tools for quality assessment of RCT study, and finally assigned a judgment of high, low, or unclear risk of material bias for each study. Our NOS analysis for observational studies demonstrated that three studies had high quality scores of ≥ 7 [9–11] with one study having a moderate score [15] (Table 3). Cochrane bias risk tool for assessing RCTs demonstrated that all four were high-quality studies (Supplementary Figure S1).

**Figure 1:**
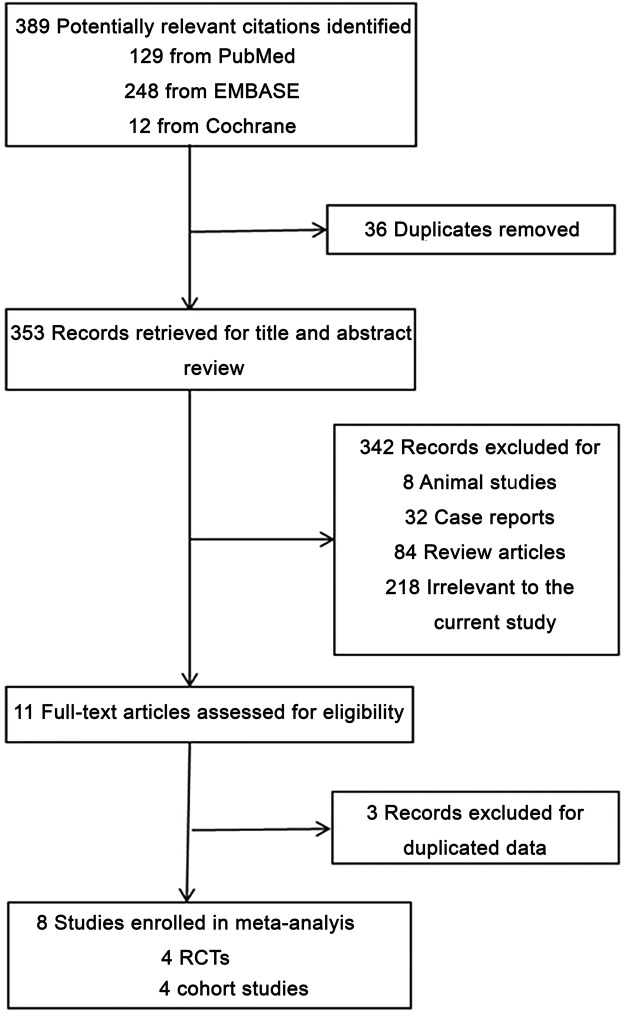
Flow diagram of study selection process

### Data extraction

Two reviewers extracted data from the eligibility studies according to standard data extraction forms. We extracted and analyzed odd ratios (ORs)/HRs/RRs values and the corresponding 95% confidence intervals (CIs) to evaluate the association between steroids and AF recurrence after RFCA. At the same time, the following data were also collected from each study: study characteristics (first author’s last name, year of publication, origin of the studied population, study population, number of patients, follow-up duration, diagnosis and detection methods of AF recurrence, ablation, dose and type of steroids in the steroids group) and patient characteristics (age, male, percentage of paroxysmal AF, hypertension, diabetes, heart failure, antiarrhythmic, echocardiographic parameters, the procedure time of catheter ablation, superior vena cava (SVC) isolation). Any disagreements between the two reviewers (M.L. and M.G.) were resolved by discussion with a senior reviewer (T.L.).

### Statistical analysis

Review Manager (version 5.3) was used for statistical analysis. *P*<0.05 was considered to be statistically significant. Categorical data were described in percentages and continuous variables were reported as mean and S.D. Results of AF recurrence after RFCA were presented as OR and 95% CI. Heterogeneity was evaluated with Chi-square tests and *I^2^*, and *I^2^* ≥ 50% was considered to indicate significant heterogeneity. If *I^2^* < 50% then a fixed-effects model was used, otherwise the random-effects model was used. Subgroup analyses were based on study design (cohort study and RCT study).

## Results

### Patient baseline characteristics

A flow diagram detailing the search and study selection process is illustrated in Figure 1. Three hundred and eighty-nine citations were initially retrieved from PubMed, Embase, and the Cochrane online database. Thirty-six records were discarded as duplicates**.** Subsequent records including 8 animal studies, 32 case reports, 84 review articles, and 218 irrelevant studies were also excluded accordingly. Subsequently, we excuded 3 records [16–18] of duplicated data by reading 11 full-text articles. In the end, a total of eight studies involving 992 patients between 2010 and 2016 met our selection criteria for inclusion [8–15]. The baseline characteristics of all patients are summarized in Tables 1 and 2, respectively. Seven of eight studies [8–11,13–15] examined outcomes of AF patients undergoing RFCA. The follow-up period varied from 6 to 38 months. It is worth noting that different steroids, including hydrocortisone, dexamethasone, prednisone, and methylprednisolone were used in these studies. Furthermore, the steroid administration method also varied between articles.

**Table 1:** Characteristics of studies included in the meta-analysis

First author, year	Design	Country	Study population	Number of patients (*n*) S/C	Follow-up (month)	The variety of steroids	Dosage and use time	Ablation	Diagnosis of AF recurrence	Methods of AF detection
Krishnan, 2010 [15]	Cohort study	U.S.A.	AF who underwent RFCA	37/31	NR	Dexamethasone	Iv 11.9 ± 4.6 mg/day during hospitalization	NR	Any AF > 10 min during hospitalization	Continuous electrocardiographic monitoring
Kim Y.R., 2012 [12]	RCT	NR	NR	56/56	3	Methylprednisolone	Iv 0.5 mg/kg for post-procedural day 1 and orally 12 mg/day for post-procedural days 2–5	NR	Atrial tachyarrhythmia	Holter monitoring
Won, 2013 [11]	Cohort study	Korea	Drug-resistant AF who underwent RFCA	89/120	12	Hydrocortisone	Iv 100 mg within 30 min after RFCA	3.5-mm irrigated-tip catheter, 50°C, 25–35 W	Any episode of AF or AT of at least 30 s	Holter monitoring ECGs
										Intensive questioning regarding any arrhythmia related
Andrade, 2013 [10]	Prospective cohort study	Canada	Drug resistant AF who underwent RFCA	45/45	12	Hydrocortisone	Iv 250 mg after transseptal access	3.5-mm irrigated-tip catheter, 43°C, 30–35 W, 17–30 ml/min, on the posterior wall, 20–25 W, 17 ml/min	Symptomatic electrocardiographically documented AF or AFL, or AT, at least ≥ 30 s	Routine transtelephonic Monitoring Clinical assessment Holter monitoring ECGs
Koyama, 2010 [8]	RCT, DB	Japan	Drug resistant AF who underwent RFCA	60/65	14	Hydrocortisone and prednisonlone	Iv hydrocortisone 2 mg/kg in the day of PVI and orally prednisonlone 0.5 mg/kg/day for 3 days after PVI	8-mm distalelectrode 52°C, 30–35 W	ECG showed AF, irrespective of symptoms	Holter monitoring ECGs Intensive questioning regarding any arrhythmia related Electrocardiographic monitoring
Kim D.R., 2015 [9]	Cohort study	Korea	Drug resistant AF who underwent RFCA	L 95/ M 97/95	24	Low: hydrocortisone Moderate: methylprednisolone	Low: Iv 100 mg within 30 min after RFCA Moderate: Iv 125 mg within 30 min after RFCA	3.5-mm irrigated-tip catheter 50°C, 25–35 W	Any episode of AF or AT of at least 30 s	Holter monitoring ECGs
Kim Y.R., 2015 [13]	RCT, DB	Korea	AF who underwent RFCA	64/74	24	Methylprednisolone	Iv 0.5 mg/kg before the femoral vein puncture and orally 12 mg/day for the following 4 days	3.5-mm irrigated-tip catheter 43°C, 30 W	Any episode of AF/AFL/AT of at least 30 s	ECGs Telemetry monitoring Handheld ECG device Holter monitoring
Iskandar, 2016 [14]	RCT	U.S.A.	AF who underwent RFCA	30/30	12	Prednisone	Orally three doses of 60 mg Q day prior to the procedure	NR	NR	Event monitoring

Abbreviations: AT, atrial flutter; DB, double blind; ECG, electrocardiogram; Iv, intravenous injection; NR, not reported; PVI, pulmonary vein isolation; S/C, steroid group/control group.

**Table 2:** Patients’ characteristics of studies included in the meta-analysis

First author, year	Age (years), S/C	Male S/C *n* (%)	Paroxysmal AF, S/C, *n* (%)	Hypertension S/C, *n* (%)	Diabetes S/C, *n* (%)	HF S/C, *n* (%)	Antiarrhythmic drugs, S/C, *n* (%)	
							Class Ic	Class III
Krishnan, 2010	55 ± 10*	51 (75)*	38 (56)*	NR	NR	8 (12)*	NR	NR
Kim Y.R., 2012	NR	NR	NR	NR	NR	NR	NR	NR
Won, 2013	55 ± 11/55 ± 11	70 (78)/94 (78)	50 (56)/57 (48)	38 (45)/52 (44)	4 (5)/16 (14)	2 (2)/4 (3)	53 (60)/70 (58)	27 (30)/45 (38)
Andrade, 2013	58.4 ± 9.5* (57.4 ± 10.5/59.5 ± 8.3)	33 (72)/31 (69)	45 (100)/45 (100)	11 (24)/16 (36)	0 (0)/3 (7)	NR	NR	NR
Koyama, 2010	60.7 ± 9.6* (59.8 ± 8.7/61.5 ± 10.3)	48 (80)/52 (80)	60 (100)/65 (100)	36 (55.4)/29 (44.6)	NR	NR	41 (68.3)/39 (60.0)	23 (38.3)/33 (50.8)
Kim D.R., 2015	L56 ± 9/M56 ± 10/56 ± 10	L80 (84)/M82 (84)/80 (85)	L58 (63)/M61 (64)/60 (63)	L46 (48)/M49 (52)/40 (42)	L6 (6)/M13 (14)/10 (10)	L3 (3)/M5 (5)/2 (2)	L61 (64)/M61 (63)/54 (57)	L23 (24)/M30 (31)/39 (41)
Kim Y.R., 2015	56 ± 10* (56 ± 9/56 ± 10)	51 (79.7)/50 (67.6)	138*	21 (32.8)/27 (36.5)	8 (12.5)/10 (13.5)	8 (12.5)/3 (4.1)	43 (68.4)/54 (73.3)	7 (10.5)/16 (22.2)
Iskandar, 2016	63 ± 7*	NR	60 (100)*	NR	NR	NR	NR	NR

**Table 2:** Patients’ characteristics of studies (continued)

First author, year	Catheter ablation, S/C			SVC isolation S/C, *n* (%)	Echocardiographic parameters, S/C		
	Total duration of procedure, min	Total fluoroscopytime, min	Duration of RF ablation, min		LAD, mm	LVDED, mm	LVEF, %
Krishnan, 2010	NR	NR	NR	NR	40.7 ± 6.8*	NR	NR
Kim Y.R., 2012	NR	NR	NR	NR	NR	NR	NR
Won, 2013	188 ± 45/201 ± 52	47 ± 21/48 ± 16	82 ± 28/89 ± 32	4 (5)/7(6)	43 ± 5/41 ± 6	50 ± 4/50 ± 4	63 ± 7/63 ± 8
Andrade, 2013	176 ± 49* (169 ± 35/182 ± 60)	39 ± 16* (35 ± 8/42 ± 20)	53 ± 20* (58 ± 21/48 ± 19)	NR	38.0 ± 5.7* (37.5 ± 5.8/38.5 ± 5.6)	NR	NR
Koyama, 2010	205.1 ± 34.3* (200.8 ± 35.6/209.1 ± 32.6)	81.4 ± 24.4* (81.4 ± 22.3/82.6 ± 24.3)	48.1 ± 17.6* (47.9 ±15.8/48.2 ± 19.3)	4 (6.6)/3 (4.6)	38.3 ± 7.4* (37.6 ± 7.1/38.9 ± 7.7)	48.3 ± 4.9* (48.4 ± 5.3/48.3 ± 4.5)	64.4 ± 8.8* (66.1 ± 7.3/62.8 ± 9.8)
Kim D.R., 2014	L182 ± 46/M168 ± 40/192 ± 52	L58 ± 19/M43 ± 13/48 ± 16	L77 ± 27/M68 ± 23/86 ± 24	L2 (2)/M14 (15)/6 (7)	L41 ± 5/M42 ± 7/42 ± 6	L49 ± 5/M50.6 ± 4/52 ± 8	L64 ± 7/M62 ± 9/61 ± 6
Kim Y.R., 2015	377 ± 97* (363 ± 94/390 ± 99)	37 ± 8* (37 ± 9/38 ± 8)	123 ±38* (118 ± 38/127 ± 38)	6 (9.4)/8 (10.8)	41.6 ± 6.2* (40.9 ± 5.8/42.2 ± 6.5)	142.5 ± 33.7* (142.2 ± 37.7/142.7 ± 30.9)	59.5 ± 6.6* (57.8 ± 8.7/ 60.5 ± 6.1)
Iskandar, 2016	NR	NR	56 ± 10.65/51 ± 13.53	NR	NR	NR	NR

* means the data of all populations. Abbreviations: HF, heart failure; LAD, left atrial diameter; LVDED, left ventricular end-diastolic dimension; LVEF, left ventricular ejection fraction; NR, not reported; S/C, steroid group/control group.

**Table 3:** NOS quality evaluation for cohort studies

First author, year	Selection				Comparability	Outcome			Total
	Representativeness of the exposed cohort	Selection of the non-exposed cohort	Ascertainment of exposure	Demonstration that outcomes were not present at the start of study	Comparability on the basis of the design or analysis	Assessment of outcome	Adequate follow-up duration	Adequacy of follow-up of cohorts	Score
Krishnan, 2010	★	★	★	★	0	0	0	★	5
Won, 2013	★	★	★	★	★ (age, sex) ★ (paroxysmal AF)	0	★	★	8
Andrade, 2013	★	★	★	★	★ (age, sex) ★ (paroxysmal AF)	0	★	★	8
Kim D.R., 2015	★	★	★	★	★ (age, sex) ★ (paroxysmal AF)	★	★	★	9

### Efficacy of steroids in preventing AF recurrence post-RFCA

As the impact of steroids on AF recurrence after RFCA were reported at different follow-up periods, we evaluated the association between steroid use and AF recurrence post-RFCA at (i) 2–3 days, (ii) 1, (iii) 3, (iv) 12–14, and (v) 24 months, respectively. Only data from the low-dose steroid group in the Kim et al. study were used for this meta-analysis [9].

#### AF recurrence with steroids 2–3 days after RFCA

**Figure 2:**
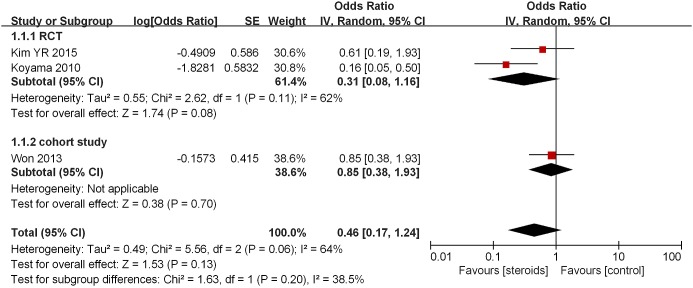
Association between steroids and AF recurrence in 2–3 days after ablation Subgroup analysis based on study design was also presented.

Three studies reported AF recurrence 2–3 days after RFCA [8,11,13]. Of these, one was an RCT that showed that steroids reduced AF recurrence [8], whereas the remaining two studies found no observable benefit [11,13]. Though insignificant, pooled analysis of the included studies demonstrated that patients treated with steroids had lower odds of AF recurrence when compared with the control group (OR = 0.46, 95% CI = 0.17–1.24, *P*=0.13; Figure 2). Similarly, subgroup analyses into two RCTs also showed that steroid use was not significantly associated with decreased risk of AF recurrence [8,13] (OR = 0.31, 95% CI = 0.08–1.16, *P*=0.08; Figure 2). The overall heterogeneity was low across subgroups (*I^2^* = 39%).

#### AF recurrence with steroids 1 month following RFCA

**Figure 3:**
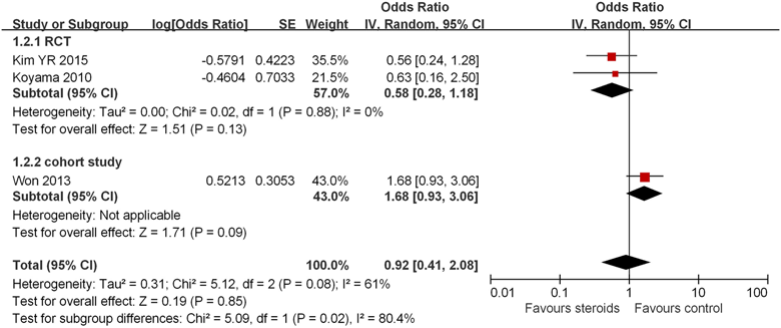
Association between steroids and AF recurrence in 1 month after ablation Subgroup analysis based on study design was also presented.

Three studies reported AF recurrence 1 month after RFCA [8,11,13]. Our analysis showed that the use of steroids post-RFCA is not associated with an increased risk of 1-month AF recurrence (OR = 0.92, 95% CI = 0.41–2.08, *P*=0.85; Figure 3). Subsequent analyses based on study design also revealed that steroid therapy was not associated with AF recurrence in 1 month after RFCA, whether in RCTs [8,13] (OR = 0.58, 95% CI = 0.28–1.18, *P*=0.13; Figure 3) or in the cohort study [11] (OR = 1.68, 95% CI = 0.93–3.06, *P*=0.09; Figure 3). However, the heterogeneity was found to be high across subgroups (*I^2^* = 80%). To locate the origin of the heterogeneity, sensitivity analysis excluding one study at a time was performed. Doing so did not significantly alter the overall heterogeneity.

#### AF recurrence with steroids 3 months following RFCA

**Figure 4:**
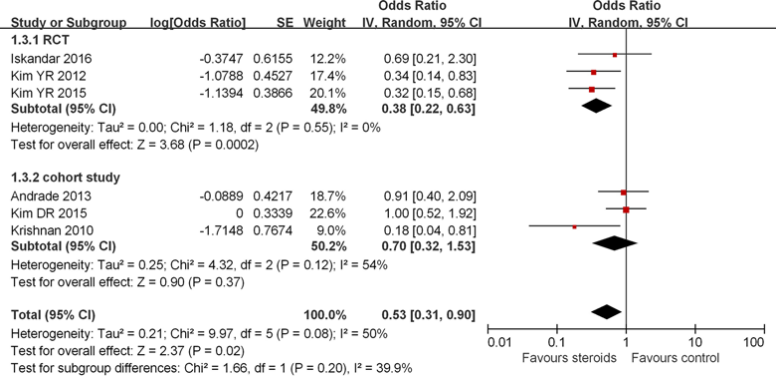
Association between steroids and AF recurrence in 3 months after ablation Subgroup analysis based on study design was also presented.

Six studies reported AF recurrence 3 months after RFCA [9,10,12–15]. Of these, three studies [12,13,15] showed that steroids use reduced the risk of AF recurrence, whereas the remaining three studies [9,10,14] did not find any significant benefit. The pooled analysis of the included studies demonstrated that patients treated with steroids have lower odds of AF recurrence 3 months after RFCA when compared with controls (OR = 0.53, 95% CI = 0.31–0.90, *P*=0.02; Figure 4). When further subgroup analyses were conducted, steroid use was associated with decreased risk of AF recurrence in RCTs [12–14] (OR = 0.38, 95% CI = 0.22–0.63, *P*=0.0002; Figure 4) but not in cohort studies [9,10,15] (OR = 0.70, 95% CI = 0.32–1.53, *P*=0.37, Figure 4). The heterogeneity was low across subgroup (*I^2^*= 40%).

#### AF recurrence with steroids 12–14 months following RFCA

**Figure 5:**
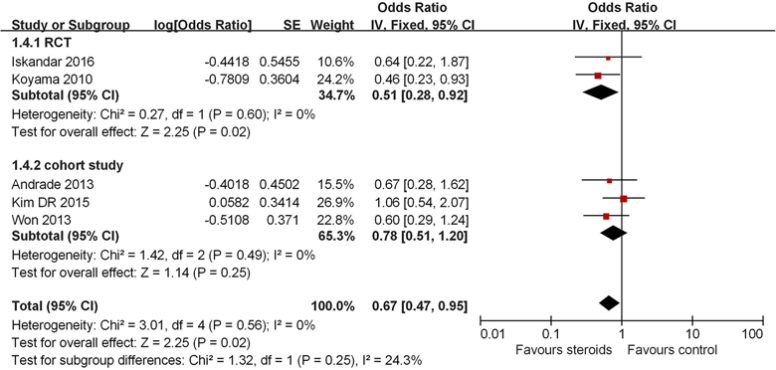
Association between steroids and AF recurrence in 12–14 months after ablation Subgroup analysis based on study design was also presented.

Five studies reported AF recurrence 12–14 months after RFCA [8–11,14]. Of these, one study [8] demonstrated decreased risk of AF recurrence with the use of steroids with the remaining studies reporting a lack of benefit. Nevertheless, the pooled analysis demonstrated reduced odds of AF recurrence with steroid use (OR = 0.67, 95% CI = 0.47–0.95, *P*=0.02; Figure 5). Once again, subgroup analyses demonstrated statistically significant reduction in AF recurrence in RCTs [8,14] (OR = 0.51, 95% CI = 0.28–0.92, *P*=0.02, Figure 5), but not in cohort studies [9–11] (OR = 0.78, 95% CI = 0.51–1.20, *P*=0.25, *I^2^* = 0%; Figure 5). The heterogeneity was low across subgroups (*I^2^* = 24%).

#### AF recurrence with steroids 24 months following RFCA

Only one study [13] reported AF recurrence 24 months after RFC but steroid use was not associated with significant difference in AF recurrence whether in univariate (HR = 1.03, 95% CI = 0.58–1.81, *P*=0.922) or multivariate analysis (HR = 0.97, 95% CI = 0.55–1.71, *P*=0.918).

## Discussion

The main findings of this systematic review and meta-analysis are that steroid use is associated with a decreased risk of AF recurrence 3 and 12–14 months after RFCA. On the other hand, there seems to be no significant association between steroid therapy and risk of AF recurrence in 2–3 days, 1 and 24 months after RFCA.

However, we found a significant heterogeneity, which could have been attributed to the type of AF (paroxysmal/persistent), intensity of ECG monitoring on follow-up, lesion sets deployed, doses of steroids used, and the time points on follow-up at which recurrences were evaluated. This heterogeneity is likely responsible for the conclusion that short-term steroid use decreases AF recurrence at 3 and 12 months, but not at 2–3 days and 1 month. Effective doses or dosing intervals to decrease AF recurrence are not fully established, even in cardiac surgery studies.

Inflammation induced by ablation can promote acute AF recurrence [19], which plays an important role during this period, increasing oxidative stress, promoting fibrosis, and facilitating re-entry [20–23]. It has been demonstrated that colchicine is an effective and safe agent for preventing early AF recurrences after pulmonary vein isolation in patients with paroxysmal AF, associated with reduced CRP and IL-6 levels [24]. The use of steroid to prevent AF has mainly been evaluated in cardiothoracic surgery. Previous meta-analyses have reported that steroid treatment is associated with a decrease in more than 50% postoperative cases [25]. The use of steroids is beneficial in this regard given their anti-inflammatory properties. Indeed, the levels of inflammatory cytokines immediately post-ablation were lower in the steroid group compared with the control group [14]. Moreover, CRP levels 3 days after ablation were lower in the corticosteroid group [8,9]. Furthermore, Koyama et al. [8] reported that AF recurrence 1 month after RFCA was not related to changes in inflammatory markers such as CRP. However, factors other than CRP in the pro-inflammatory signaling cascade can mediate pro-fibrotic changes. Given the main role of inflammation in immediate and early AF recurrence after ablation, treatments targetting this process may have a great potential to ameliorate this adverse event improving prognosis. Steroids can inhibit pro-inflammatory response, suppress VEGF expression and inhibit fibroblast proliferation [26]. These effects can modulate the pro-arrhythmic substrate and reduce conduction defects [27].

The major cause of longer term recurrence may be associated with electrical reconnection between PVs and LA after apparently successful initial isolation. The occurrence of this reconnection results from deficiencies of the index ablation procedure [26]. Rizzo et al. observed that α-lipoic acid (ALA) therapy could reduce inflammation stress, but could not prevent AF recurrence at 12-month follow-up after catheter ablation [28]. In this study, we found that steroid therapy decreased the risk of AF recurrence in 12–14 months after RFCA. However, it seems fairly implausible that steroid use shortly after PVI could prevent re-conduction between the LA and PVs over the long term. First, structural and electrical remodeling takes place within a few hours of AF onset, whereas reverse remodeling after restoration of sinus rhythm occurs much more slowly [29]. The use of steroids may halt electrical or functional remodeling of the atria, thereby permitting reverse remodeling to occur. Second, AF also induces inflammation, which in turn can perpetuate AF [30]. Steroid therapy shortly after RFCA might halt the relationship between inflammation and AF, which may represent a vicious cycle. Third, gaps of sufficient cross-sectional dimensions within linear RF lesions may promote recovery of conduction [31]. Steroids can potentially prevent AF recurrence by suppressing the delayed extension of the RF lesion between the ablation points [12].

Indeed, Andrade et al. [10] suggested that steroids use remained independently associated with the high prevalence of dormant PV conduction unmasked by adenosine. Therefore, a balance of anti-inflammatory action and delayed extension of the RF lesion determines the net pro- or anti-arrhythmic effect of steroids. Moreover, ablation can lead to transient autonomic dysfunction by increasing the sympathetic tone and decreasing the parasympathetic tone [32]. These could promote arrhythmias by both triggered activity and re-entry [33].

## Study limitations

Several potential limitations of this meta-analysis should be acknowledged. First, our analysis pooled together both RCTs and cohort studies, but subgroup analyses based on study design were also performed. Second, our study involved a relatively small number of subjects (*n*=992). Third, the moderate heterogeneity observed was only partially explained by study design, and other factors such as steroid dose may play a role. Steroid dosing was not differentiated in most of the included studies, it was therefore not possible to derive dose–response relationships between steroids and AF recurrence after ablation. Fourth, in most of studies, although we identified AF recurrence by using the ECG and Holter monitoring during follow-up, some asymptomatic intermittent AF events might have been missed. Fifth, less than one-third women were included in the studies. Thereby, data may not be applicable to female patients.

## Conclusion

Our meta-analysis suggested that steroid use was associated with a decreased risk of early AF recurrence 3 and 12–14 months after ablation. No clear relationship was observed for 2–3 days, 1 and 24 months of follow-up. Further data are needed to clarify the clinical merit of this intervention in the long term, and provided the dose information of steroid for prevention of AF recurrence.

## Supporting information

**Supplementary Figure 1:** Cochrane bias risk tools of RCTs A. Risk of bias graph. B. Risk of bias summary.

## Abbreviations

AF: atrial fibrillation
CI: confidence interval
CRP: C-reaction protein
HR: hazard ratio
LA: left atrium
IL-6: interleukin 6
NOS: Newcastle–Ottawa scale
OR: odd ratio
PV: pulmonary vein
RCT: randomized clinical trial
RF: radiofrequency
RFCA: RF catheter ablation
RR: relative risk
VEGF: vascular endothelial growth factor
