# Supplementary material for: Steroids prevent early recurrence of atrial fibrillation following catheter ablation: a systematic review and meta-analysis
Source: Biosci Rep. 2018 Oct 15;38(5):BSR20180462. doi: 10.1042/BSR20180462 (PMC6435499; doi:10.1042/BSR20180462)

Supplementary Figure 1. Cochrane bias risk tools of RCTs A. Risk of bias graph. B. Risk of bias summary.

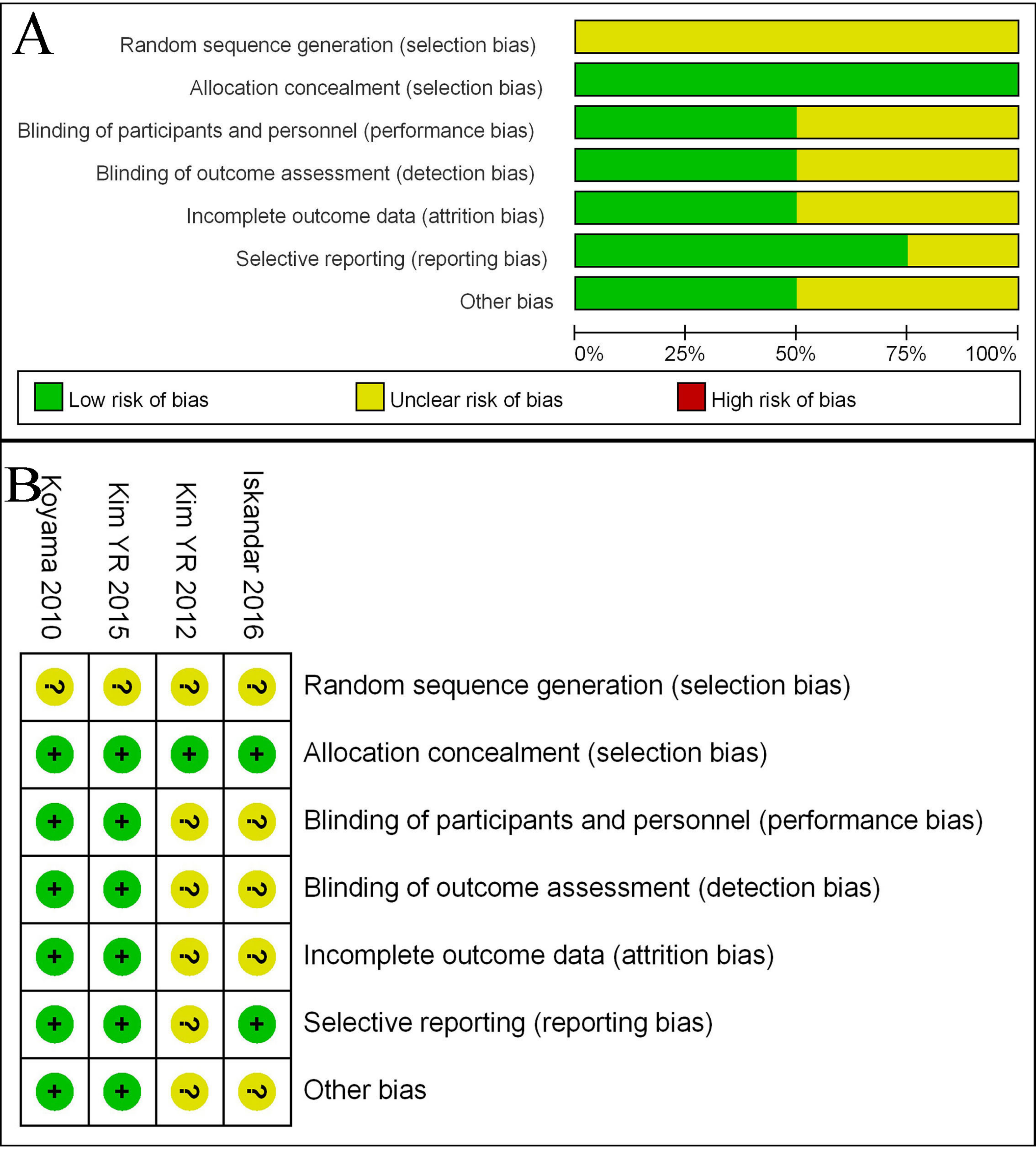

Supplement: Supplementary file 1 [file bsr20180462_Supp1.pdf]
